# Intelligence, Correspondence, &c.

**Published:** 1826-01

**Authors:** 


### INTELLIGENCE, CORRESPONDENCE, &c.

*Royal Medical Hall,*
*Edinburgh, 22d. Nov,* 1825.

Sir,

You will confer a favour on the Royal Medical Society of Edinburgh by inserting in your publication the following paragraph.

The Royal Medical Society of Edinburgh proposes, as the subject of their Prize Essay, the following questions.

1st. What is the respective agency of the veins and the lymphatics in the process of absorption?

2nd. By what means or mechanism do these vessels accomplish this process? What are the proofs which shew that the substances absorbed, are taken up by open mouths or orifices, or pass through the coats in the manner of imbibition or transudation?

3rd. Is there any reason to believe that the individual animal tissues possess a distinct power of absorption, or, that this process is influenced by the nature of the animal tissues?

The sum of Twenty Guineas, a Medal, or a set of books of that value will be given to the Author of the best Dissertation on the Subject proposed by the Society, for which all men of science are invited to compete.

The dissertation may be written in English, French, or Latin, and must be transmitted to the Secretary, on or before the first of December, 1826, and the adjudication of the prize will take place in the last week of the month of February following.

To each dissertation must be prefixed a motto, which must likewise be written on the outside of a sealed packet, containing the name and address of the author. No dissertation will be received with the Author's name affixed; and all dissertations, except the successful one, will be returned, if desired, with the sealed packet unopened.

NICOLSON BAIN, Secretary.

---

We are requested to insert the following Notice in our Journal.

In the 2nd edition of Beck's Elements of Medical Jurisprudence, published in London by Mr. Dunlop, page 104, and in a note at the foot of the page, is the following passage.

"The facts (relating to the trial of Angus for Murder) from which the above case has been prepared, are drawn from a review of the trial of Mr. Angus, and the pamphlet, to which it gave rise, in the Edinburgh Med. and Surg. Journal, vol. v p. 220; also a pamphlet, entitled 'A Vindication of the Opinions delivered, in evidence, by the Medical Witnesses for the Crown, on a late Trial at Lancaster for Murder.' Liverpool, 1808. *This masterly production is from the pen of Dr. Bostock*"

This last sentence contains a very great mistake. The pamphlet abovementioned was not from the pen of Dr. Bostock. It was written by Dr. Rutter: and the mistake would have been corrected sooner if it had been sooner observed.

Again, at page 103, it is asserted that "three cases are related by Dr. Bostock, to whom they were communicated by Mr. Kendrick, of Warrington, of the Disease under consideration, &c." (Hydatids.) Here is another mistake. The Cases were communicated by Dr. Kendrick to Dr. Rutter, by whom they were mentioned in the above pamphlet.

And again, at page 142, it is said that Dr. Bostock states, that "the complete contraction (of the uterus) seldom takes place in less than 18 days, and it is often more." Now, as Dr. Bostock did not write the pamphlet alluded to, it is obviously an error to say that he had made such a statement.

If these remarks should fall under the observation of the author of the Elements of Jurisprudence, or of the English Editor of them, it is but right to expect that they will, in all future editions of the work, correct the mistakes here pointed out to them; and if there be auy merit in the pamphlet abovementioned, common justice requires that it should be rendered to him to whom, on this occasion, it strictly and alone belongs.

---

### OBITUARY.

The Profession has to lament the loss of Mr. JOHN BROUGH TAYLOR, of Sunderland, Member of the Royal College of Surgeons in London, who died of fever on the first day of October, 1825, in the 39th year of his age. His abilities were respectable, and his attainments considerable. He had a taste as well as talent for natural history, and was preparing for publication a work entitled "*Flora Fossilis,*" which was patronized by several distinguished subscribers. Whether in professional consultation, or social converse, he was affable and gentlemanly, and, as a husband and father, friend, or member of society, his character was amiable and exemplary. He ranked among his warm friends many estimable and talented individuals, who respected him living, and regret him dead. His premature death excites the most melancholy feelings, from the consciousness of his merit, and the recollection of his having left an affectionate and intelligent widow, and three children, who are too young to be sensible of that great misfortune, by which their comforts may be abridged, and their prospects clouded.

### NOTICE TO CORRESPONDENTS.

Many favours have been unavoidably postponed till next Number. Among these we may acknowledge (from Dr. B——T) the Review of Dr. Good's new edition of the Study of Medicine, which was not received till the 1st December, long after the Review Department was closed. It will certainly appear in the April Number. From Dr. H——n we have received several articles for the Periscope, from the Foreign Journal which he has undertaken to superintend; but have only been able, in this Quarter, to give place to one or two, which he will find near the close of the ANALECTA MINORA. The others in our next.

From Dr. K. many papers have been received, among which we may here acknowledge the Analysis of Dr. Dill's "*Dissertatio Inauguralis de Inhalatione per cutem*," which shall find a place in our next. Also the article on Auscultation, embracing an extensive review of Dr. Stokes' work on that subject. From our able Correspondent, Mr. J. we have received the review of Dr. Stokes' Stethoscopical Publication, and must endeavour to amalgamate the two Reviews of the same subject in our Number for April.

To Mr. S. of B. we are under many obligations, and he will see some of his favours in the present Number.

Our Somerton Correspondent will see an account of Mr. Bryce's instrument in the present Number. We cannot speak from personal experience of its merits; we have reason to think it will prove inferior to the Stomach-pump, chiefly in its weak extractive (syphonic) powers. We do not wish, however, to prejudice the public against a cheap substitute for the comparatively expensive patent machines of Weiss and Read, provided it be found useful.

To several Correspondents who have asked how it is that they cannot procure Sir Astley Cooper's work on Dislocations, &c. We can only say that a new edition is reported to be in the Press. We would recommend the worthy Baronet to print a very large Edition at once, and thus supply the public demand.

Several Subscribers have enquired for a Review of Dr Parry's Posthumous Work. They will see their wishes complied with in the present Number. A full account of the Posthumous Writings of Dr. Baillie will appear in our next, with Commentaries. They will not bear to be published, *in toto*, without doing injustice to the purchasers of this Journal. Veneration for the dead must not supersede duty to the living.

### *Oil of Euphorbia Lathyrus.*

This important medicine has been at length imported into this country, and may be had at Mr. Noakes', (successor to the late Mr. Pope) 96, Oxford-street. The medicine is, at present, dearer than we were led to believe, but it is likely to prove invaluable, having all the good, and none of the bad qualities, of croton oil. Dose, from 3 to 8 drops—taste, as insipid as almond oil.

OBSTETRIC SOCIETY.

An Association of this kind has been formed in the Metropolis on a broad and liberal scale, which promises to be of much service to the obstetric branch of the profession, and to society at large. One grand object of the Society is, ultimately to procure a legislative enactment for the regulation of obstetric practitioners, both male and female. In the mean time, the Society is to consist of three classes—resident members (physicians, surgeons, and apothecaries, practising midwifery)—non-resident (country) members, of the same description—and honorary (but efficient and contributing) members, including physicians and surgeons who *have*, and who have *never* practised midwifery. There is to be a house, library, museum —weekly and monthly meetings—and a monthly gazette of the Society's transactions.

The officers for the first year—a President, (Charles Mansfield Clarke) a Vice President, (Dr, Merriman) and ten Council. Among the officers are Drs. Armstrong, Johnson, Kerrison, Thomson, Ramsbottom, Granville, &c.; Messrs. Blagden, Stone, North, &c. We shall give a more detailed plan in our next. Mean time, we have no doubt that gentlemen who wish well to the undertaking will quickly enrol their names, (if in town) or transmit them (if in the country) to any of the gentlemen mentioned above —or (post paid) to the Editor of this Journal.

---

JOHN BARON, M.D. FRS. has in the press, Delineations of the Origin and Progress of various Changes of Structure which occur in Man and some of the inferior Animals, being the continuation of Works already published on this subject by the Author. The Work will be handsomely printed in quarto, and will contain highly finished coloured Plates, after accurate Drawings.

---

DR. REECE has in the press, a Practical Treatise on the Means of Obviating and Treating the Varieties of Costiveness at different Periods of life, and in cases of Predisposition to various constitutional Maladies, and of Disorders of the Lungs, Stomach, Liver, Rectum, &c. by Medicine, Diet, &c. In one vol. 8vo.

---

*Velpeau on the Spinal Marrow.** This writer infers, from experiments made by himself and others, that, under certain circumstances, the spinal marrow may be severely injured, cut, interrupted, nay, destroyed for a considerable extent, without occasioning death or even the perceptible alteration of any function; a conjecture which he believes to stand opposed to every thing previously taught or known respecting the uses of the nervous system.

He advances, not without diffidence, the following hypothesis as an explanation of the facts which he relates. 1st, That though the different parts of the nervous system are mutually connected with each other, they are, to a certain degree, capable of exercising their functions independently of each other. 2nd, That though sensation and motion be naturally dependant on the principal nervous trunk, yet, should this be wanting in any part, a communication of nervous influence may be kept up between the upper and lower portions, by means of the lateral branches. 3rd. That, in the instances which he has related of interruption of the spinal marrow, innervation is in the point corresponding to these interruptions by means of the

---

* See Article 1st in the Pathological Division of this compensated Periscope.

branches which connect the nerves immediately on their exit from the spinal canal.

---

*On the Reproduction of the Crystalline Lens.*—M. Coquehean has laid before the Royal Academy of Medicine of Paris, a paper, designed to prove, from experiments made on young animals, such as puppies and young rabbits, that the crystalline lens may be reproduced, provided it be extracted with care, and with the least possible injury to its capsule. If such be the fact, it is quite accordant with the opinion maintained by Professor Blainville respecting the nature of the lens, and the office of its capsule.

---

*Langier, on a concretion taken from the human intestine, and erroneously supposed to resemble the collections of hair called egager piles which are found in the ox and some other animals.*—This specimen was extracted from the rectum. A fragment of bone formed the nucleus, the major part of it consisted of vegetable matter, and it was covered by a thin layer of animal matter, which appeared to be little else than dried blood. M. Langier attributes its formation to a habit of chewing liquorice or other substances capable of affording minute particles of woody fibre.

This concretion seems to be very closely allied to those occasionally formed in the intestines of persons of the lower orders in Scotland, and which, we believe, were first examined by Dr. Duncan, jun. and subsequently by Dr. Wollaston and Dr. Thompson, and which have been clearly shewn to be derived from the downy or pubescent part of the oat.

---

*Insensibility of the Retina.* At the Meeting of the Academy of Sciences, on the seventeenth of January, Magendie related the following fact.—Having occasion to operate on the eye, he accidentally touched the retina, when he remarked, that by doing so, he did not appear to produce any sensation, he then retouched it, intentionally, and still without causing any sensation.

---

*The sense of Smell not dependant on the first pair of Nerves.* The late Professor Beclard mentioned a case at one of the sittings of the Academy of Medicine in February, which seemed to support the ideas of those physiologists, who are of opinion that the first pair of nerves do not preside over the sense of smell, but, that this office must be assigned to a branch of the fifth pair. On inspecting the body of a man, who, to the latest moment of his life appeared to retain the faculty of smell, it was found that the first pair of nerves, together with the inferior portion of the anterior lobes of the brain were in a carcinomatous state. A similar case was long since observed by Mercy, and stands recorded in the memoirs of the Academy of Sciences.

---

MIDDLESEX INFIRMARY, *No 37, Great Pulteney Street, Golden Square.*—This Institution, which was established for special objects, (the diseases of women and children, and midwifery) has been extended and remodelled for the general purposes of a dispensary. The medical practice will be under the direction of Dr. James Johnson, and another physician—the surgical department will be under James Boyle, Esq. and another surgeon—while that of midwifery will be superintended by George Jewel, Esq.

It is expected that, by the ensuing winter, this institution will be rendered available for the instruction of medical pupils in the West End of the town. The physicians and surgeons will take especial pains to convey to the pupils in attendance every species of clinical information in their power, both as regards the investigation and the treatment of diseases. More particulars will be stated in our next.

