# Errata in Last Number

**Published:** 1826-04

**Authors:** 


### *Errata in last Number.*

Page 302—line 10 from top, *for egager piles, read egagropiles,* and *for Langier, read Laugier* in the preceding line.

